# Application of the Er:YAG laser in pulpotomy for mature permanent teeth with pulpitis: An animal study

**DOI:** 10.1371/journal.pone.0341017

**Published:** 2026-01-30

**Authors:** Zeqi Li, Pengfei Xin, Siyao Yang, Xiaoxing Hao, Jun Wang, Kuanshou Zhang, Qingmei Liu

**Affiliations:** 1 Shanxi Medical University School and Hospital of Stomatology, Taiyuan, China; 2 Department of Stomatology, Third Hospital of Shanxi Medical University, Shanxi Bethune Hospital, Shanxi Academy of Medical Sciences, Tongji Shanxi Hospital, Taiyuan, China; 3 State Key Laboratory of Quantum Optics and Quantum Optics Devices, Institute of Opto-Electronics, Shanxi University, Taiyuan, China; University of Puthisastra, CAMBODIA

## Abstract

**Objective:**

To apply Erbium-doped Yttrium Aluminum Garnet (Er:YAG) laser in pulpotomy for experimentally induced pulpitis in mature permanent teeth of rats, and investigate whether Er:YAG laser can improve the effect of pulpotomy in mature permanent teeth with pulpitis.

**Methods:**

Thirty-six 3-month-old male Sprague-Dawley (SD) rats were selected, with bilateral maxillary first molars as experimental teeth. 4 rats (8 experimental teeth) were selected to construct the pulpitis model. After confirming the successful establishment of the model by this method, 32 rats were randomly divided into the control group and the experimental group, and the pulpitis models were constructed in the same way. The control group(mechanical group, 16 rats, 32 experimental teeth) received pulpotomy with conventional sterile excavators. The experimental group (laser group, 16 rats, 32 experimental teeth) received pulpotomy using Er:YAG laser. At 3, 7, 14, and 28 days post-operation, four rats from each group were randomly euthanized. Pathological changes in the dental pulp were observed using hematoxylin - eosin (HE) staining. Immunohistochemical (IHC) assessment and mean optical density (MOD) measurement were performed to evaluate the expression of interleukin-1β (IL-1β) and partitioning defective protein 3 (Par3). Inter-group differences were analyzed using the Mann-Whitney U test or the independent samples t-test.

**Results:**

Histological examination by HE staining demonstrated favorable pulpal repair in the laser-treated group. The total histopathological scores were significantly lower in the laser group compared to the mechanical group at days 3 and 7 post-operation (*p* < 0.05). However, no statistically significant difference was observed between the two groups at days 14 and 28. IHC analysis revealed that the mean optical density (MOD) values for IL-1β were consistently lower in the laser group at all four time points (*p* < 0.05), while the MOD values for Par3 were consistently higher in the laser group (*p* < 0.05).

**Conclusion:**

The Er:YAG laser used during pulpotomy in mature permanent rat teeth with pulpitis preserves the remaining healthy pulp tissue, reduces IL-1β expression, and enhances Par3 expression, thereby alleviating inflammation and promoting tissue repair.

## Introduction

Pulpitis refers to the inflammatory response of the dental pulp tissue to various harmful stimuli, including biological, physical, chemical, and other factors, which usually progresses from a reversible stage to an irreversible stage. Previously, we believed that when bacteria invaded the dental pulp tissue, it indicated that pulp inflammation had progressed to histologically irreversible pulpitis [1]. However, the European Society of Endodontology (ESE) stated in 2021 that current clinical examination methods cannot accurately diagnose the pulp inflammation status, nor determine the relationship between the pulp inflammation status and its healing potential [2]. Ricucci et al.. have proposed that although there is local pulp necrosis in the area invaded by bacteria, the radicular pulp tissue remains normal, and the pulp still has the potential for repair [3]. These literatures have all posed challenges to the contemporary nomenclature of irreversible pulpitis. Traditionally, vital pulp therapy(VPT) has been primarily indicated for teeth diagnosed with reversible pulpitis. However, according to contemporary guidelines [4,5], for mature permanent teeth with symptomatic irreversible pulpitis, VPT can also serve as an important treatment option after strict case selection and exclusion of contraindications.

Pulpotomy is one of the vital pulp therapies, referring to the removal of infected pulp while retaining the remaining healthy pulp. Depending on whether only the superficially affected tissue or the entire coronal pulp is removed, the technique is classified as either partial pulpotomy or full pulpotomy. Currently, pulpotomy is primarily used to treat pulpitis in primary teeth and immature permanent teeth, aiming to protect the developing permanent tooth germ and promote apical development and closure of the apical foramen [6]. iRoot BP Plus is a new nanobioceramic material that has been gradually used in clinical practice in recent years. It is mainly composed of calcium silicate, zirconia and calcium hydroxide, which have good physical and chemical properties and biological compatibility.A number of current studies have shown the following [7,8]: Compared with the traditional pulp capping agent calcium hydroxide, iRoot BP Plus has a shorter curing time and slightly expands during curing, which is conducive to the formation of a good seal with dentin. Moreover, it can induce the proliferation and differentiation of dental pulp stem cells into odontoblasts, thereby promoting the formation of reparative dentin.

Therefore, with the continuous improvement in the performance of pulp capping agents and the in-depth research on the biology of dental pulp, an increasing number of scholars have attempted to perform pulpotomy in mature permanent teeth with pulpitis [9–12].Compared with root canal therapy, pulpotomy has the advantages of simple operation, short treatment time, low cost, low technical sensitivity, and a high long-term retention rate of the affected teeth.This suggests that teeth that have traditionally been considered inaccessible for endodontic restoration can be treated by removing infected and inflamed tissues, thereby maintaining the vitality of the remaining pulp and the health of the periapical tissue. However, after summarizing and analyzing some of the failed cases [13,14], it was found that poor control of infected microorganisms was one of the main causes of failure. If the sterility of instruments such as digging spoons is not guaranteed during operation,disinfection and sterilization can not be achieved, and external infected microorganisms might also be brought to the pulp section.

In many experiments and some clinical operations [15–17], researchers have applied the Er: YAG laser to preserve vital pulp in primary teeth and young permanent teeth, which can cut pulp tissue in a non-contact state and sterilize the pulp section at the same time. The application of lasers has greatly improved the success rate of aseptic operation, and avoided the risks of pulp infection and mechanical damage that may be caused by pulp amputation using a spoon excavator. The wavelength of the Er: YAG laser is 2940 nm, and most of the energy emitted by it is absorbed by water and hydroxyapatite, causing a “micro-explosion”, which breaks the target tissue and cuts the tissue. Moreover, Er: YAG laser can be absorbed by water molecules in the dental pulp tissue, which vaporize quickly and take away a large amount of heat, with little damage to the tissue below the pulp section [18]. In addition, the excellent hemostatic effect of the Er: YAG laser can cause the pulp capping agent to be in close contact with the pulp section [19].

Currently, the application of Er:YAG laser in the treatment of mature permanent tooth pulpitis is limited to isolated case reports, with a paucity of basic research evidence. The null hypothesis (H₀) of this study is: In the treatment of mature permanent tooth pulpitis in rats, Er:YAG laser-assisted pulpotomy and traditional mechanical (excavator) pulpotomy exhibit no significant difference in the resolution of pulp inflammation postoperatively. To test this hypothesis, the present study intends to apply Er:YAG laser to pulpotomy for mature permanent tooth pulpitis in rats. Through pathological sectioning and immunohistochemical analysis, we will systematically compare the effects of the two techniques on the resolution of pulp inflammation, tissue repair, and the expression of relevant molecules. The aim is to elucidate the underlying mechanism of action of Er:YAG laser, thereby providing experimental evidence for the clinical application of Er:YAG laser-assisted vital pulp preservation technology.

## Materials and Methods

### Grouping of experimental rats

**Fig 1:**
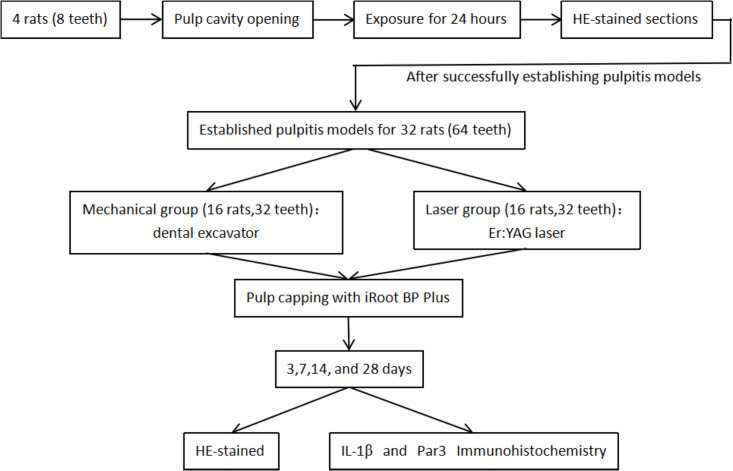
Experimental flow chart of this study.

All animal experiments were conducted in strict compliance with Laboratory Animal—Guideline for Ethical Review of Animal Welfare (GB/T 35892−2018), issued by the Standardization Administration of the People’s Republic of China. The protocol was approved by the Institutional Animal Care and Use Committee (IACUC) of Shanxi Bethune Hospital/Shanxi Academy of Medical Sciences (No. SBQDL-2022–126). A total of 36 specific-pathogen-free (SPF) male Sprague-Dawley rats, aged 3 months, were selected. All rats weighed 430 ± 10 g, exhibited intact permanent dentition, and were free from caries, malformations, and oral diseases. Four rats were randomly selected from this group as the Pulpitis Model Validation Group. Their bilateral maxillary first molars served as experimental teeth (8 teeth total). This preliminary phase aimed to validate the method for inducing experimental pulpitis in mature permanent teeth. A sample size of 32 rats was determined through Power Analysis and Sample Size (PASS) software (Version 21, NCSS, LLC, Utah, USA), based data from previous literature [20], ensuing a minimum power of 0.8, α = 0.05, and accounting for a 5% dropout rate. Using the simple randomization method, an independent researcher who was not involved in the subsequent experiment generated random numbers via the GraphPad QuickCalcs online randomization tool. These numbers were then assigned as unique identifiers to each rat, which were further randomly allocated to either the control group or the experimental group. The grouping information was recorded, sealed, and stored. After establishing pulpitis models using identical methods in all rats, the control group (also termed the mechanical group; 16 rats, 32 teeth) underwent pulpotomy with a conventional sterile excavator. The experimental group (also termed the laser group; 16 rats, 32 teeth) received Er:YAG laser-assisted pulpotomy. At 3, 7, 14, and 28 days post-operation, four rats from each group were randomly selected and euthanized for sample collection. All subsequent procedures—including HE staining, immunohistochemistry, and statistical analysis—were performed by evaluators blinded to group assignments. The overall experimental workflow is summarized in Fig 1.

### Model establishment and validation

Pulpitis was induced using a pulp chamber exposure method. Rats were placed in an anesthesia induction chamber and anesthetized with 4% isoflurane (RWD Life Science, shenzhen, China) delivered in an oxygen/air mixture at a flow rate of 1 L/min. Subsequently, intraperitoneal injection of 4% sodium pentobarbital (40 mg/kg; Solarbio, beijing, China) was administered to maintain anesthesia. A smooth, blunt mouth prop was used to retract the oral cavity and expose the maxillary first molars. The surgeries were performed under aseptic conditions. Each rat was operated on using an independent set of autoclaved instruments. The maxillofacial surgical site, as well as the teeth and oral mucosa, were disinfected three times with 75% ethanol. Access opening was performed under a dental operating microscope (OMS2380, ZUMAX, Suzhou, China, magnification: 4x-25x) by a senior endodontist. A round FG1/4 bur (MANI, Utsunomiya, Japan) was used with water spray to prepare a Class I cavity in the central fossa of the occlusal surface. Drilling continued until a pinkish hue became visible through the thin residual dentin. Pulp chamber was penetrated using a DG16 probe (Dentsply Maillefer, Balligues, Switzerland), with slight bleeding indicating successful penetration. Hemostasis was achieved by applying a saline-moistened cotton pellet for 2–3 minutes. The pulp chamber was left open.

Based on previous literature [21–23], 24 hours after pulp exposure to the oral environment, HE-stained sections of rat molars show extensive inflammatory cell infiltration near the pulp access cavity and necrotic foci limited to the coronal pulp. The radicular pulp tissue remained morphologically relatively healthy, and no typical histopathological features of bacterial infection were observed. This histologic condition is deemed suitable for pulpotomy in the present study. Therefore, a 24-hour exposure period was selected for establishing the pulpitis model.

According to the AVMA Guidelines [24], rats were euthanized by an intraperitoneal overdose injection of sodium pentobarbital (150 mg/kg). The maxillae containing bilateral first molars were immediately dissected. Successful induction of experimental pulpitis was confirmed through HE staining Kit (Solarbio, beijing, China).

### Experimental procedures

**Fig 2:**
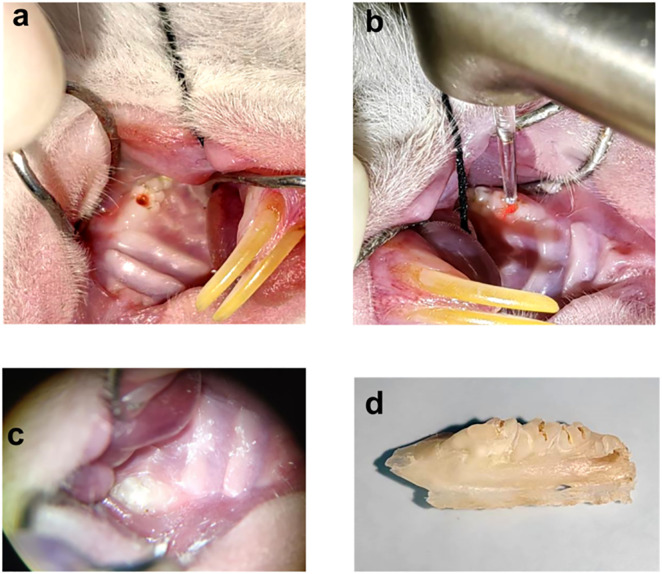
Representation of the experimental operation: (a) The pulpitis model was constructed by access opening. **(b)** The pulpotomy was performed using Er:YAG laser. **(c)** After the pulpotomy under the microscope. **(d)** Maxillary bone specimen.

After inducing pulpitis as described, pulpotomy was performed in both the mechanical and laser groups. Rats were anesthetized using the same method previously described. All procedures were carried out under a dental operating microscope by a senior endodontist. Necrotic debris around the pulp access cavity of the maxillary first molars was removed using a sterile excavator (Guangdong Kehui Medical Technology Co., Ltd., Yangjiang, China). In the mechanical group, a new sterile excavator was used to remove the coronal pulp to the level just above the root canal orifices. To avoid interference from the oxidizing properties of sodium hypochlorite with subsequent molecular biological assays, we irrigated the pulp chamber with normal saline in the experiment. Hemostasis was achieved by applying compression with a saline-moistened cotton pellet for 2–3 minutes. In the laser group, an Er:YAG laser (LightWalker AT(S), Fotona, Germany) with handpiece No. 69082 was used. Coronal pulp amputation was performed using the following parameters: Pulse mode: SWEEPS (double-pulse sequence, 25 µs each), 30 mJ, 20 Hz, 0.6 W, energy density: ≈ 2 J/cm². The fiber tip was held 2 mm from the pulp tissue under air-water spray cooling. Pulp amputation lasted approximately 10 seconds. After normal saline irrigation, hemostasis was achieved using the Er:YAG laser with the following parameters: Pulse mode: SP (single short pulse, 300 µs), 30 mJ, 15 Hz, 0.45 W, energy density: ≈ 1 J/cm².The fiber tip was positioned 5 mm from the tissue and applied for 5 seconds. All laser parameters were selected based on previous studies demonstrating similar tissue effects and fell within the manufacturer’s recommended ranges [25–27]. Following pulpotomy, iRoot BP Plus (Innovative BioCeramix Inc., Vancouver, BC, Canada) was applied to completely cover the amputated pulp surface in both groups. The material was placed to a thickness of approximately 1 mm. The light-cured glass ionomer cement (Fuji II LC,GC, Tokyo, Japan) was then placed, followed by dental adhesive (3M Single Bond™, 3M ESPE Dental Products, St. Paul, MN, USA) application. The cavity was restored with flowable resin composite (3M Filtek Z350 Flow, 3M ESPE Dental Products, St. Paul, MN, USA). To minimize the risk of restoration loss, the filling was adjusted to eliminate occlusal contact. Postoperatively, animals were housed singly in an individually ventilated cage (IVC) system. The animals’ mental state, activity, food intake, and wound condition were observed and recorded daily. On postoperative days 3, 7, 14, and 28, four rats from each group were euthanized for specimen collection. Part of the operation diagram is shown in Fig 2.

### Hematoxylin-eosin (HE) staining

Rats were euthanized as scheduled. The maxillae containing the bilateral first molars were promptly dissected. Restorative materials were carefully removed from the pulp chambers. Specimens were fixed with fixative solution (Servicebio, Wuhan, China), decalcified with decalcifying solution (Solarbio, Beijing, China), and then embedded in paraffin wax (Beyotime, Beijing, China). Serial sections were cut mesiodistally along the long axis of the tooth, and six 4-μm-thick sections were prepared from each specimen. Three of the most representative consecutive sections, which contained the central cross-section of the pulp exposure site and the adjacent intact coronal pulp tissue, were selected for subsequent analysis: one section was subjected to HE staining for histological scoring, while the remaining two sections were processed for immunohistochemical staining targeting IL-1β and Par3, respectively. A scoring system for evaluating the inflammatory status of dental pulp was established in the present study (Table 1), with reference to the grading protocols reported in previous literature [28,29]. Two specialists in dental pulp pathology performed histopathological observation and scoring of the dental pulp sections in a blinded manner to the experimental group allocation.

**Table 1:** Scores used during the histological examination: inflammatory cell response, tissue disorganization, and reactionary dentin formation.

Score	Characterization		
Inflammatory cell response	Tissue disorganization	Reactionary dentin formation
0	No or only a few scattered inflammatory cells are visible on the pulp wound surface	Normal dental pulp tissue	Formation of thick and continuous hard tissue deposition
1	Inflammatory cell infiltration confined to the vicinity of the pulp wound surface	Pulp tissue disorganization confined to the vicinity of the wound surface	Formation of thin and continuous hard tissue deposition
2	Inflammatory cell infiltration involving most of the coronal pulp	Pulp tissue disorganization involving most of the coronal pulp	Formation of scattered hard tissue deposition
3	Inflammatory cell infiltration involving the entire coronal pulp and even extending to the radicular pulp	Pulp tissue disorganization involving the entire coronal pulp and even extending to the radicular pulp	Absence

### Immunohistochemistry (IHC) staining

Following baking, deparaffinization, and rehydration, antigen retrieval was performed. Sections were incubated at 4°C overnight with Rabbit Anti-IL-1β polyclonal antibody (1:200; ab283818, Abcam, Cambridge, UK) or Rabbit Anti-Par3 polyclonal antibody (1:100; bs-9510R, Bioss Biotechnology, Beijing, China). The next day, sections were rinsed with PBS and incubated with a secondary antibody at room temperature for one hour. The DAB substrate (Abcam, Cambridge, UK) was applied, and color development was monitored under a light microscope (ZEISS, Oberkochen, Germany). The reaction was terminated when brown-yellow staining became visible. After counterstaining, acidification, blueing, dehydration, and mounting, a technician blinded to the experimental groups captured images under high magnification (×200). Mean optical density (MOD) was measured using Image-Pro Plus 6.0 software (Media Cybernetics, Inc., Rockville, MD, USA), and results are expressed as mean ± standard deviation (x̄± s).

### Statistical analysis

Inter-rater reliability analysis was performed using SPSS software (version 27, IBM Corp., Armonk, NY, United States) to calculate the intraclass correlation coefficient (ICC) of the histological scores assigned by the two pathologists. Intergroup comparisons were conducted with GraphPad Prism software (version 10, GraphPad Software Inc., La Jolla, CA, USA): the Mann-Whitney U test was used for histopathological score comparisons; normality and homogeneity of variances of the mean optical density (MOD) values of IL-1β and PAR3 were assessed, and independent samples t-test (or Mann-Whitney U test) was applied accordingly. A P-value < 0.05 was considered statistically significant. The statistical analysis was conducted under blinded conditions by one researcher using anonymized data (identified solely by rat numbers). An independent verification of the entire statistical output was then performed by another researcher. After completion of the analysis, rat IDs were matched with group information to produce the final results.

## Results

### Construction of a rat pulpitis model

**Fig 3:**
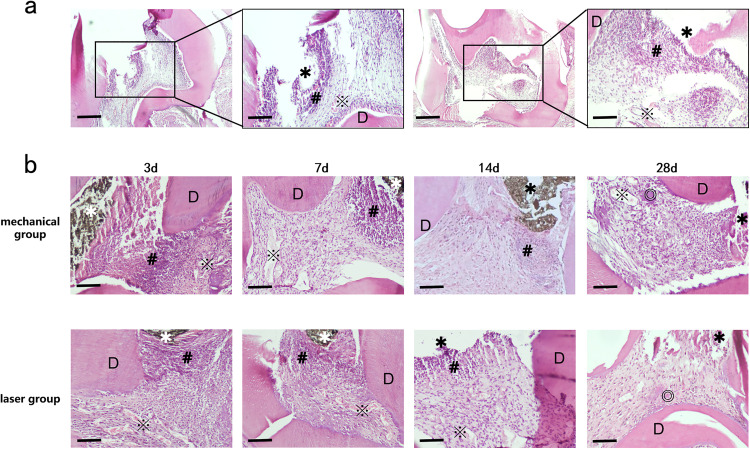
(a) HE staining (×40) and local magnification (×100) histological images represent dental pulp tissue after 24 hours of access opening. **(b)** HE staining histological images represent the pulp tissue of mechanical group and laser group at 3d, 7d, 14d and 28d after pulpotomy(×100). Scale bar = 200 μm. ✱ pulp access cavity (The dark brown substance at the pulp access cavity was identified as residual pulp capping material); D dentin; # Inflammatory cells; ※ Capillaries; ◎ Hyperplastic calcified tissue.

As shown in Fig 3a, the pulp access cavity—created to expose the pulp chamber—is visible. The most severe inflammatory response was localized around this access cavity, indicating the epicenter of pulpitis. This pulp access cavity also served as the entry point for subsequent pulpotomy procedures.

HE staining showed that necrotic and exudative tissue were centered around the pulp access cavity 24 hours after pulp exposure. The adjacent odontoblast layer appeared disorganized. Significant inflammatory cell infiltration was observed beneath the necrotic area, extending approximately halfway through the coronal pulp. Dilated capillaries were visible beneath the zone of inflammation. These findings confirm successful induction of pulpitis. The same model preparation procedure was followed for all subsequent pulpotomy experiments.

### Morphological observation of rat dental pulp tissue

Throughout the experimental follow-up period, all rats included in the study remained in good condition, and no unexpected deaths occurred. Fig 3B presents representative HE-stained sections of maxillary first molars from the mechanical and laser groups after pulpotomy. In the mechanical group, day 3 showed necrotic tissue beneath the pulp access cavity, accompanied by intense infiltration of neutrophils and other inflammatory cells. Disorganized pulp cells and mildly dilated capillaries were observed deeper in the tissue. By day 7, the inflammatory infiltration had reduced in depth, pulp cell arrangement improved, and noticeably dilated capillaries appeared near the canal orifices alongside scattered inflammatory cells. On day 14, inflammatory cells were further reduced and formed limited focal aggregates. At day 28, minimal necrotic and exudative material remained near the access opening without significant inflammatory infiltration. Dilated capillaries and clustered reparative calcifications were evident in deeper areas.

In the laser group, day 3 exhibited limited necrotic debris under the pulp access cavity with inflammatory cells infiltrating deeper zones. Multiple dilated capillaries were visible near the pulp chamber floor. By day 7, inflammatory infiltration depth had decreased, and dilated capillaries were observed close to the root canal orifices. On day 14, inflammatory cells continued to reduce with persistent dilated capillaries below. At day 28, minimal exudate and scattered inflammatory cells were present under the pulp access cavity. Scattered reparative calcifications were observed near the pulp chamber floor and root canal orifices. No significantly dilated capillaries were identified.

Two specialists in endodontic pathology evaluated the histopathological morphology of the aforementioned dental pulp sections while blinded to the experimental groups. The scores assigned by the two evaluators demonstrated a high degree of consistency (intraclass correlation coefficient, ICC > 0.90). Statistical analysis based on these scores revealed that the laser group exhibited significantly superior histological status compared with the mechanical group at 3 and 7 days post-treatment (*p* < 0.05). whereas no statistically significant difference was detected in the total histological scores between the two groups at 14 and 28 days post-treatment. The total histological scores of the two groups are presented in Table 2.

**Table 2:** Total histological scores at different time points after pulpotomy in the two groups (M (Q1-Q3)).

	3d	7d	14d	28d
Mechanical	8.250 (7.250-8.500)	5.500 (5.000-6.250)	4.250 (3.625-4.875)	3.250 (3.000-3.875)
Laser	6.250 (6.000-7.875)	5.000 (4.125-5.000)	3.000 (3.000-4.125)	3.000 (2.000-3.500)
*p*	< 0.05	< 0.05	> 0.05	> 0.05
95% CI	−2.500 ~ −0.500	−2.000 ~ 0.000	−1.500 ~ 0.000	−1.500 ~ 0.500
r_rb_	0.75	0.67	0.53	0.25

### IHC staining of IL-1β

**Fig 4:**
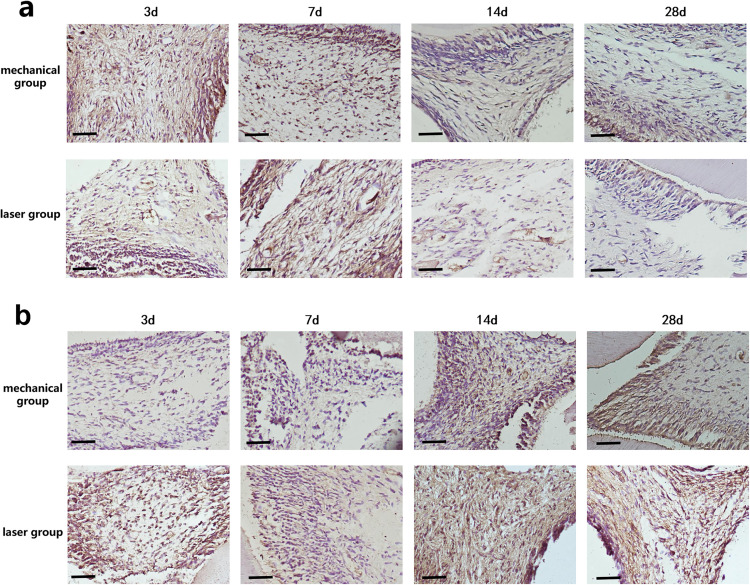
(a) IHC staining images represent both groups of IL-1β levels at 3d, 7d, 14d and 28d after pulpotomy (×200). **(b)** IHC staining images represent both groups of Par3 levels at 3d, 7d, 14d and 28d after pulpotomy (×200).Scale bar = 100μm.

Fig 4a presents the immunohistochemical staining results for IL-1β in both the mechanical and laser groups. The corresponding mean optical density (MOD) values are summarized in Table 3. Within each group, IL-1β expression significantly decreased over time following pulpotomy (*p* < 0.05). As shown in Table 3, the laser group exhibited significantly lower IL-1β expression than the mechanical group on days 3, 7, and 14 post-operation (*p* < 0.05). By day 28, IL-1β expression markedly decreased in both groups. However, the laser group still showed significantly lower expression compared to the mechanical group (*p* < 0.01).

**Table 3:** MOD values of IL-1β positive expression at different time points after pulpotomy in the two groups (x̄ ± s).

	3d	7d	14d	28d
Mechanical	47.06 ± 5.90	37.48 ± 7.65	27.23 ± 3.00	19.56 ± 3.00
Laser	38.36 ± 5.93	28.21 ± 6.31	20.46 ± 6.18	14.26 ± 2.80
*p*	< 0.05	< 0.05	< 0.05	< 0.01
95% CI	−15.04 ~ −2.36	−16.79 ~ −1.75	−11.98 ~ −1.56	−8.41 ~ −2.19
η²	0.3820	0.3330	0.3567	0.4880

### IHC staining of Par3

Fig 4b shows the immunohistochemical staining for Par3 in the mechanical and laser groups. The corresponding mean optical density (MOD) values are presented in Table 4. Within each group, Par3 expression increased significantly over time after pulpotomy (*p* < 0.05). As illustrated in Table 4, the laser group showed significantly higher Par3 expression than the mechanical group on days 3, 14, and 28 (*p* < 0.05). On day 7, Par3 expression remained significantly elevated in the laser group compared to the mechanical group (*p* < 0.01).

**Table 4:** MOD values of Par3 positive expression at different time points after pulpotomy in the two groups (x̄ ± s).

	3d	7d	14d	28d
Mechanical	14.22 ± 1.86	21.22 ± 5.74	38.21 ± 5.91	62.22 ± 4.95
Laser	20.18 ± 5.73	35.91 ± 8.52	45.04 ± 4.50	69.97 ± 7.00
*p*	< 0.05	< 0.01	< 0.05	< 0.05
95% CI	1.40 ~ 10.52	6.90 ~ 22.48	1.20 ~ 12.46	1.25 ~ 14.25
η²	0.3590	0.5387	0.3256	0.3186

## Discussion

Traditionally, the detection of bacterial invasion in dental pulp tissue has been regarded as a histological hallmark of irreversible pulpitis, and is directly associated with an unfavorable prognosis [30]. However, with the deepening understanding of the biological potential of the pulp-dentin complex, we have come to recognize that the dental pulp is more capable of resisting microbial invasion than previously thought. During the progression of dental caries and pulpitis, the pulp-dentin complex triggers a series of repair mechanisms, including the release of bioactive factors [31,32], formation of sclerotic dentin [33], and initiation of programmed inflammatory responses [34]. However, how to maximize the protection and mobilization of this intrinsic potential by optimizing treatment techniques remains a current research focus. By comparing laser and mechanical pulpotomy, this study aims to evaluate the effects of these two interventions with distinct properties on the key indicators of pulpal inflammation regulation and reparative dentin formation postoperatively, thereby providing experimental evidence for the clinical selection of minimally invasive methods that are more consistent with the biological characteristics of the dental pulp.

The purpose of this study was to test the following null hypothesis (H₀): There is no significant difference in the pulp tissue response after pulpotomy between Er:YAG laser and traditional mechanical methods. However, our results do not support this hypothesis. HE staining of pulp tissue sections provides direct visualization of inflammatory status and repair processes. Comparative analysis between the mechanical and laser groups revealed superior pulpal repair outcomes in the laser group at each time point. These included markedly reduced necrotic exudate, narrower inflammatory cell infiltration, and significantly improved cellular organization. Jayawardena et al. [35] reported that after direct pulp capping following accidental exposure during cavity preparation, the Er:YAG laser group exhibited significantly greater reparative dentin formation at one and two weeks compared to the dental handpiece group, which is consistent with our findings. Takamori [36] observed enhanced proliferation of pulp fibroblasts and increased reparative dentin deposition in Er:YAG-lased mouse molars at day 14 relative to the dental handpiece group; however, no significant differences were noted between the groups at days 21 and 35. This experimental design focused on comparing the therapeutic effects of the two surgical interventions, and thus neither pre-operative time points nor a sham surgery group were included. This limitation restricts the evaluation of the absolute magnitude of changes induced by the treatments. However, existing literature has demonstrated that in analogous models, the sham surgery group fails to achieve substantial inflammatory resolution and dentin bridge formation, exhibiting therapeutic efficacy far inferior to that of the standard positive control group [37,38]. Furthermore, by designating the current standard approach as the positive control, we ensured the validity of relative comparisons between groups.

IL-1β is a core pro-inflammatory cytokine involved in acute inflammatory responses, which exacerbates tissue destruction by promoting the synthesis of matrix metalloproteinases (MMPs) and other pathways [39,40]. However, a growing body of evidence indicates that in pulpitis, short-term exposure to IL-1β or its presence at low concentrations can enhance the odontogenic differentiation of dental pulp stem cells (DPSCs) and improve their mineralization capacity, suggesting a potential positive role of IL-1β in the repair phase following inflammation control [41,42]. In the present study, IL-1β expression levels in both groups were observed to gradually decrease over time after pulpotomy, with a concomitant improvement in the inflammatory status of the dental pulp. This dynamic change suggests that the elevated IL-1β in the early postoperative period may be involved in the necessary initiation of inflammation, while its subsequent decline marks the resolution of the pro-inflammatory state, thereby creating a favorable environment for the tissue to transition into a repair-dominant phase. A systematic review [43] reported a 97.4% clinical success rate at 12 months and 93.97% at 36 months after pulpotomy in teeth with signs and symptoms of irreversible pulpitis. These results support the feasibility of VPT in mature permanent teeth with pulpitis, which aligns with the present findings. Comparatively, the laser group showed significantly lower IL-1β expression than the mechanical group at all time points, suggesting better anti-inflammatory and reparative outcomes. Zhao et al. [44] observed markedly reduced IL-1β expression after direct pulp capping using low-level laser therapy combined with Dycal in rat molars. Their results indicate that laser treatment may suppress IL-1β to exert anti-inflammatory effects, consistent with the outcomes of this study.

Par3 is a key polarity protein involved in regulating cell division and polarization. It forms a conserved polarity complex with Par6 (Partitioning defective protein 6) and aPKC (atypical protein kinase C) [45,46]. This complex anchors at cell junctions to establish and maintain epithelial polarity and tight junctions [47]. In many cancers, Par3 influences biological behaviors such as proliferation and migration by modulating intercellular connections, which contributes to tumor development [48–50]. Studies have shown that Par3 is widely expressed in odontoblasts [51]. Under bacterial or toxic stimulation, odontoblasts downregulate Par3 to disrupt intercellular junctions, promoting proliferation, migration, and suppressing apoptosis. This enhances pulp repair and facilitates rapid formation of reparative dentin, preventing further bacterial invasion. These findings indicate that lower Par3 levels correlate with more severe inflammation, while its expression increases as inflammation subsides. Therefore, in this study, we assessed Par3 membrane expression as an indicator of pulpal inflammatory status. In this study, Par3 expression increased over time in both groups following pulpotomy. The laser group exhibited higher Par3 expression than the mechanical group at all corresponding time points. These results indicate that Er:YAG laser-assisted pulpotomy promotes odontoblast proliferation and differentiation more effectively than conventional mechanical methods, leading to accelerated repair of pulp tissue.

This study employed a completely randomized design instead of a within-subjects control design. Although this approach requires a larger sample size to control for individual variability, it can avoid cross-interference between different therapeutic methods and ensure inter-group independence. The expression of IL-1β and PAR3 was evaluated using immunohistochemical semi-quantitative analysis. This approach was chosen due to the extremely limited volume of rat molar pulp tissue, which makes extracting sufficient and homogeneous protein for ELISA or Western blot analysis technically challenging and prone to sampling variability. Although immunohistochemistry has inherent limitations in providing absolute quantification, it enables in situ analysis on tissue sections. By combining standardized image analysis with statistical comparison, this method allowed for a reliable assessment of relative expression differences between the two experimental groups. Future studies involving tissue protein extraction could further validate and extend the findings presented here.

### Limitations

This study provides a theoretical basis for the application of laser in pulpotomy of mature permanent teeth. However, when translating its conclusions into clinical practice, a key premise must be considered: In this study, the localized state of pulpitis (where inflammation did not involve the entire coronal pulp) was artificially controlled in the animal model, thereby ensuring the health of the remaining pulp tissue under experimental conditions. In clinical practice, however, accurately assessing the actual extent of pulp inflammation constitutes a common core challenge determining the success of all pulpotomies, including laser-assisted procedures. Currently, such assessment relies primarily on a comprehensive analysis of medical history, symptoms, signs, and limited auxiliary examinations. Its accuracy remains to be verified and optimized through larger-scale clinical trials and long-term follow-up studies.

## Conclusions

This study confirms that Er:YAG laser-assisted pulpotomy is an effective method for treating pulpitis in mature permanent teeth using a rat model. Compared with traditional mechanical methods, laser treatment exhibits greater advantages in preserving vital pulp and promoting inflammatory resolution. Its beneficial effects may be associated with a more significant reduction in IL-1β levels to control inflammation, coupled with enhanced localization of Par3 on the odontoblast membrane, which facilitates the maintenance of cell polarity. This study provides experimental evidence supporting Er:YAG laser as a more promising technical alternative in pulpotomy.

## Supporting information

S1 Table Histological scores of the Mechanical group and the Laser group assessed by Rater 1 and Rater 2. (PDF)

S2 Table MOD values of IL-1β and Par3 positive expression. (PDF)
