# Supplementary material for: Application of the Er:YAG laser in pulpotomy for mature permanent teeth with pulpitis: An animal study
Source: PLoS One. 2026 Jan 30;21(1):e0341017. doi: 10.1371/journal.pone.0341017 (PMC12858013; doi:10.1371/journal.pone.0341017)
Supplement: S1 Table — (PDF) [file pone.0341017.s001.pdf]

S1 Table: Histological scores of the Mechanical group and the Laser group assessed by Rater 1 and Rater 2

Histological scores (Rater 1)					
			Mechanical group		
	ID	Inflammatory cell response	Tissue disorganization	Reactionary dentin formation	Total score
3d	1	2	3	3	8
	2	2	3	3	8
	3	3	3	3	9
	4	2	2	3	7
	5	2	3	3	8
	6	3	3	3	9
	7	2	2	3	7
	8	2	3	3	8
7d	1	1	1	3	5
	2	1	1	3	5
	3	1	2	3	6
	4	1	1	3	5
	5	1	1	3	5
	6	2	2	3	7
	7	1	2	3	6
	8	1	1	3	5
14d	1	0	1	3	4
	2	0	0	3	3
	3	0	1	3	4
	4	0	0	3	3
	5	1	1	3	5
	6	1	1	3	5
	7	1	1	3	5
	8	0	1	3	4
28d	1	0	0	2	2
	2	0	0	3	3
	3	0	0	3	3
	4	0	1	3	4
	5	1	1	2	4
	6	0	0	3	3
	7	0	1	2	3
	8	1	1	3	5

Histological scores (Rater 1)					
			Laser group		
	ID	Inflammatory cell response	Tissue disorganization	Reactionary dentin formation	Total score
3d	1	2	3	3	8
	2	1	2	3	6
	3	1	2	3	6
	4	1	2	3	6
	5	2	3	3	8
	6	2	3	3	8
	7	1	2	3	6
	8	1	2	3	6
7d	1	1	1	3	5
	2	1	1	3	5
	3	1	1	3	5
	4	1	1	3	5
	5	0	1	3	4
	6	1	1	3	5
	7	0	1	3	4
	8	0	0	3	3
14d	1	0	0	3	3
	2	0	0	3	3
	3	0	0	3	3
	4	1	1	3	5
	5	0	0	3	3
	6	1	1	3	5
	7	0	0	3	3
	8	0	0	3	3
28d	1	0	1	3	4
	2	0	0	2	2
	3	1	1	2	4
	4	0	0	2	2
	5	0	0	3	3
	6	0	0	3	3
	7	0	0	2	2
	8	1	1	3	5

Histological scores (Rater 2)

Mechanical group

	ID	Inflammatory cell response	Tissue disorganization	Reactionary dentin formation	Total score
3d	1	2	3	3	8
	2	2	3	3	8
	3	2	3	3	8
	4	2	2	3	7
	5	3	3	3	9
	6	3	3	3	9
	7	2	2	3	7
	8	3	3	3	9
7d	1	1	1	3	5
	2	1	1	3	5
	3	2	2	3	7
	4	1	1	3	5
	5	1	2	3	6
	6	2	2	3	7
	7	1	1	3	5
	8	2	1	3	6
14d	1	0	1	3	4
	2	0	0	3	3
	3	1	1	3	5
	4	0	1	3	4
	5	1	1	3	5
	6	1	1	3	5
	7	0	1	3	4
	8	0	1	3	4
28d	1	0	0	2	2
	2	0	1	3	4
	3	0	0	3	3
	4	0	0	3	3
	5	1	1	2	4
	6	0	0	3	3
	7	0	1	2	3
	8	1	1	3	5

Histological scores (Rater 2)					
		Laser group			
	ID	Inflammatory cell response	Tissue disorganization	Reactionary dentin formation	Total score
3d	1	2	3	3	8
	2	1	2	3	6
	3	2	2	3	7
	4	1	2	3	6
	5	2	2	3	7
	6	2	3	3	8
	7	1	2	3	6
	8	1	2	3	6
7d	1	1	1	3	5
	2	1	1	3	5
	3	1	1	3	5
	4	1	2	3	6
	5	0	1	3	4
	6	1	1	3	5
	7	1	1	3	5
	8	0	0	3	3
14d	1	0	0	3	3
	2	0	0	3	3
	3	0	0	3	3
	4	1	1	3	5
	5	0	0	3	3
	6	0	1	3	4
	7	0	0	3	3
	8	0	0	3	3
28d	1	0	0	3	3
	2	0	0	2	2
	3	0	1	2	3
	4	0	0	2	2
	5	0	0	3	3
	6	0	0	3	3
	7	0	0	2	2
	8	1	1	3	5
