# Supplementary material for: Application of the Er:YAG laser in pulpotomy for mature permanent teeth with pulpitis: An animal study
Source: PLoS One. 2026 Jan 30;21(1):e0341017. doi: 10.1371/journal.pone.0341017 (PMC12858013; doi:10.1371/journal.pone.0341017)
Supplement: S2 Table — (PDF) [file pone.0341017.s002.pdf]

S2 Table: MOD values of IL-1β and Par3 positive expression

**MOD values of IL-1β positive expression**		
	Mechanical group	Laser group
3d	36.21	28.76
	42.18	34.27
	46.32	36.41
	46.76	38
	48.58	40.23
	48.95	43.18
	55.5	48.65
	51.98	37.38
7d	25.78	18.69
	30.61	23.33
	34.72	25.17
	36.5	27.04
	38.49	29.28
	40.23	30.08
	42.5	32.41
	51.01	39.68
14d	22.94	11.26
	24.17	15.21
	25.39	18.02
	26.62	20.07
	27.84	20.84
	29.07	21
	30.29	25.96
	31.52	31.34
28d	15.46	10.43
	16.49	11.39
	17.51	12.35
	18.54	13.3
	20.58	15.22
	21.61	16.17
	22.63	17.13
	23.66	18.09

**MOD values of Par3 positive expression**		
	Mechanical group	Laser group
3d	11.56	12.89
	12.32	13.55
	13.08	16.2
	13.84	18.85
	14.6	21.51
	15.36	24.16
	16.12	26.81
	16.88	27.47
7d	12.83	25.38
	15.21	25.57
	18.26	29.73
	20.62	34.67
	21.83	37.15
	24.22	43.09
	27.18	46.26
	29.61	45.43
14d	31.28	39.48
	32.31	40.46
	33.23	41.48
	34.36	42.51
	42.08	47.59
	43.21	48.63
	44.13	49.6
	45.08	50.57
28d	55.02	60.11
	57.24	63.22
	59.29	65.3
	61.19	67.38
	63.32	72.57
	65.18	74.64
	67.22	76.72
	69.3	79.83
